# Influence of Cytokines on HIV-Specific Antibody-Dependent Cellular Cytotoxicity Activation Profile of Natural Killer Cells

**DOI:** 10.1371/journal.pone.0038580

**Published:** 2012-06-11

**Authors:** Leia Wren, Matthew S. Parsons, Gamze Isitman, Robert J. Center, Anthony D. Kelleher, Ivan Stratov, Nicole F. Bernard, Stephen J. Kent

**Affiliations:** 1 Department of Microbiology and Immunology, University of Melbourne, Victoria, Australia; 2 Division of Experimental Medicine, McGill University, Montréal, Québec, Canada; 3 Kirby Institute, University of New South Wales, Sydney, Australia; 4 Melbourne Sexual Health Clinic, Alfred Health, Carlton, Victoria, Australia; Karolinska Institutet, Sweden

## Abstract

There is growing interest in HIV-specific antibody-dependent cellular cytotoxicity (ADCC) as an effective immune response to prevent or control HIV infection. ADCC relies on innate immune effector cells, particularly NK cells, to mediate control of virus-infected cells. The activation of NK cells (i.e., expression of cytokines and/or degranulation) by ADCC antibodies in serum is likely subject to the influence of other factors that are also present. We observed that the HIV-specific ADCC antibodies, within serum samples from a panel of HIV-infected individuals induced divergent activation profiles of NK cells from the same donor. Some serum samples primarily induced NK cell cytokine expression (i.e., IFNγ), some primarily initiated NK cell expression of a degranulation marker (CD107a) and others initiated a similar magnitude of responses across both effector functions. We therefore evaluated a number of HIV-relevant soluble factors for their influence on the activation of NK cells by HIV-specific ADCC antibodies. Key findings were that the cytokines IL-15 and IL-10 consistently enhanced the ability of NK cells to respond to HIV-specific ADCC antibodies. Furthermore, IL-15 was demonstrated to potently activate “educated” KIR3DL1^+^ NK cells from individuals carrying its HLA-Bw4 ligand. The cytokine was also demonstrated to activate “uneducated” KIR3DL1^+^ NK cells from HLA-Bw6 homozygotes, but to a lesser extent. Our results show that cytokines influence the ability of NK cells to respond to ADCC antibodies *in vitro*. Manipulating the immunological environment to enhance the potency of NK cell-mediated HIV-specific ADCC effector functions could be a promising immunotherapy or vaccine strategy.

## Introduction

The development of a safe and effective HIV vaccine is urgently needed. Traditional HIV vaccine constructs have focused on the induction of broadly neutralizing antibodies (BnAbs) and cytotoxic T-lymphocytes (CTL) [1]. However, several lines of evidence suggest that non-neutralizing HIV-specific antibodies could play an important role in preventing or controlling HIV infection. These antibodies can bind to infected cells and recruit innate immune effector cells, such as natural killer (NK) cells, to lyse infected cells through antibody-dependent cellular cytotoxicity (ADCC). A recent HIV vaccine efficacy trial based on a recombinant Canarypox virus prime and envelope protein boost showed partial protection from HIV infection, despite inducing only narrow neutralizing antibody responses and very modest CTL responses [2]. High levels of HIV-specific ADCC-competent antibodies were induced by this regimen, suggesting such responses may play a role in protective immunity [3], [4]. This idea is supported by elegant passive transfer experiments in Rhesus macaques, which demonstrate decreased effectiveness of BnAbs no longer capable of eliciting antibody constant region (Fc)-dependent ADCC responses [5]. Furthermore, ADCC responses have been associated with protective outcomes against immunodeficiency viruses [6], [7], in particular this has been observed in highly exposed seronegative intravenous drug users, HIV-infected elite controllers and vaccinated Rhesus macaques [8]–[10].

The potential effectiveness of infection-induced HIV-specific ADCC responses suggests that attempts to improve or modulate these responses could provide a therapeutic benefit or assist in protective immunity. Understanding the best mechanisms to present ADCC epitopes for immune recognition, or to improve the potency with which they activate NK cells, may be important for improving ADCC-based therapeutic or preventative strategies. Recent research has highlighted the importance of antibody specificity and the glycosylation of antibody Fc regions for driving efficient ADCC responses [11], [12]. It has also been demonstrated that NK cells are more likely to mediate ADCC if NK cell function was conferred through the interaction of inhibitory killer cell immunoglobulin-like receptors (KIR) and their major histocompatibility complex (MHC) class I (or HLA-I) ligands during the process of NK cell education [13]–[15]. Furthermore, soluble factors within plasma, such as cytokines, have been demonstrated to effect NK cell responsiveness [16] and have been associated with the rate of disease progression [17], potentially due to a synergistic effect on NK cell mediated anti-HIV ADCC.

Alterations in production and plasma/sera levels of cytokines during HIV infection are extensive. Investigators have reported increases in the ability of peripheral blood mononuclear cells (PBMC) to produce IL-4 [18] and IL-10 [19], while increased levels of soluble IL-10 [20], IL-7 [21], GM-CSF [22], and TNFα [23] have been observed in the plasma of infected individuals. Furthermore, reductions in the production of IL-12 and IL-15 have been reported in HIV-infected individuals [24], [25]. The presence of other soluble plasma factors has also been noted to be enhanced during HIV infection. Most notably, LPS levels are increased in HIV-infected individuals [26], and have been correlated with the level of immune activation and disease progression [27]. The influence of cytokines on NK cell-mediated HIV-specific ADCC has not been thoroughly studied. Such studies could ultimately lead to understanding how best to influence the immune environment to obtain optimal ADCC levels during therapeutic and/or prophylactic interventions.

NK cells are exquisitely sensitive to exogenous cytokines, which can increase or decrease multiple effector functions [16]. Most anti-HIV ADCC assays utilize whole serum or plasma that contains not only the antibody of interest but also variable levels of a suite of biologically activate cytokines. Indeed, chronic viral infections, such as HIV, substantially perturb plasma cytokine levels, which could plausibly influence the effectiveness of ADCC responses *in vivo*. Upon activation NK cells mediate a variety of effector functions, including the release of cytokines and chemokines, and degranulation to kill neighboring virus-infected cells. We hypothesized that exposure of NK cells to different exogenous cytokines prior to activation would differentially alter the effector functions mediated by activated NK cells. Furthermore, as educated NK cells are conferred with higher functional potential [28], we hypothesized that NK cells educated through KIR3DL1/HLA-Bw4 interactions would be more susceptible to the effects of exogenous cytokines than KIR3DL1^+^ NK cells from HLA-Bw6 homozygous individuals. We used a recently developed flow-based ADCC assay that measures NK cell activation (IFNγ synthesis and CD107a degranulation marker expression) to evaluate these hypotheses [29], [30]. Whole blood from HIV-uninfected healthy controls was incubated with HIV antigens and ADCC-competent plasma, serum or purified IgG from HIV-infected individuals. These incubations were done in the presence and absence of exogenous cytokines. NK cells responding to ADCC antibodies were evaluated for IFNγ production and expression of the CD107a degranulation marker.

## Methods and Materials

### Study Population

We studied ADCC induced NK cell activation responses elicited by sera samples from 32 HIV-infected subjects not on antiretroviral therapy recruited through the Kirby Institute (Sydney, Australia). Table 1 provides the demographics and clinical characteristics of these patients. Plasma and sera samples from an additional 19 HIV-infected individuals recruited from the Melbourne Sexual Health Centre and one HIV-infected individual from La Clinique l’Actuel, Montreal, Quebec, Canada were utilized to study envelope-specific ADCC responses. HIV-uninfected healthy laboratory volunteers provided whole blood, for assessment of NK cell function. All subjects provided informed consent for participating in this study and human research and ethics committees from all participating study sites approved this study.

**Table 1:** Clinical characteristics of HIV-infected donors.

	Mean (range)
**Number**	32
**Age**	41 (28–65)
**Female/Male**	1/31
**CD4^+^ cell count (cells/µl)**	746.5 (504-1310)
**Viral load (copies/ml)**	290 (<50-203100)

### HLA Typing

Kits from Atria Genetics was used to conduct sequence-based typing of HLA-B alleles. Otherwise, HLA-B typing was performed by the Victorian Transplant and Immunogenetics Service (Parkville, Australia), using sequence-based typing.

### Anti-HIV ADCC NK Cell Activation Assay

As previously described [12], a whole blood assay was used to assess NK cell activation by ADCC Abs. Briefly, 150 µl of HIV-uninfected healthy control whole blood plus 50 µl of ADCC-competent HIV-infected plasma/serum (stored at −20°C), or purified IgG, was incubated at 37°C for 5 hours with 1 µg/ml of HIV Env peptide pool or whole gp140 protein, Brefeldin A (5 µg/ml, Sigma) and Monensin (6 µg/ml, Sigma). ADCC responses were assessed using either a peptide pool containing 15-mers that overlapped by 11 amino acids or whole gp140 protein. The peptide pool spanned the HIV-1 consensus subtype B Env protein (kindly supplied by the NIH AIDS Reagent Repository). As previous described, the gp140 protein was obtained from purification of the supernatant of HeLa or 293T cells transfected with the gp140 gene from the subtype B AD8 isolate [31]. After incubation, cells were surface stained with Per-CP-conjugated anti-CD3, FITC-conjugated anti-CD2, PE-conjugated anti-KIR3DL1, PE-Cy7-conjugated anti-CD56 and APC-conjugated anti-CD107a (All from BD Biosciences). Next, whole blood was treated with lysing solution (BD Biosciences) to remove red blood cells, and the remaining white blood cells were treated with permeabilization solution (BD Biosciences) and stained with Alexa700-conjugated anti-IFNγ antibody (BD Biosciences). Flow cytometry data was collected using a FACS Canto II Flow cytometer (BD Biosciences), and was analyzed using Flow Jo Version 9.2 software (Tree Star). We have previously shown this assay is not dependent on immune complexes activating NK cells [30]. The assay works efficiently using either overlapping peptides or whole Env protein as the HIV antigen.

### IgG Purification and Depletion from Sera

To assess the impact of soluble serum/plasma factors on the skewing of NK cell activation profiles, we purified total IgG from serum over a protein G spin column (Thermo Fisher Scientific) to use in the ADCC ICS assay [12]. Sera were bound to protein G columns for 4 hours with end over end agitation before elution of IgG as per the manufacturer’s instruction. Purified IgG samples were then dialyzed in 2 ml mini dialysis tubes (GE Healthcare) before they were concentrated in 30 kDa Amicon ultra centrifugal filter devices (Millipore). IgG was also depleted from sera using protein G spin columns (Thermo Fisher Scientific). IgG-depleted sera was then combined with IgG from a single source and used in the ADCC ISC assay described above, to assess the effect of soluble sera factors on a known ADCC-mediated NK cell activation response.

### Anti-Env IgG ELISA

100 ng of HIV-1_AD8_ Env gp140 purified from media conditioned by a stable gp140-expressing cell line (31) was absorbed onto the bottom of ELISA plate wells in coating buffer (20 mM Tris pH 8.8, 100 mM NaCl) overnight at 4°C. Wells were then blocked with blocking buffer (5% skim milk powder in PBS/0.1% Tween 20) for 1 hour. Patient samples were added as half log dilution series in block buffer and incubated for 4 hours at room temperature followed by washing with PBS/0.1% Tween 20. HRP conjugated antibody against human IgG in blocking buffer was then added and incubated for 1 hour. After washing ELISAs were developed using standard techniques. Background was measured by titration of HIV negative human sera. Wells were considered positive when OD was at least 5-fold higher than background. Endpoint titers (most dilute samples giving a positive reading) were averaged over two assays.

### Cytokines

*In vitro* supplementation with the following cytokines and growth factors, for the five hour duration of the anti-HIV ADCC assay, was studied to assess their influence on NK cell activation profiles: IL-10 (50 ng/ml) (BD Biosciences), IL-15 (5 ng/ml) (R&D Systems), IL-4 (50 Units/ml) (BD Biosciences), GM-CSF (1 µg/ml) (BD Biosciences), IL-12 (100 ng/ml) (R&D Systems), IL-7 (50 ng/ml) (BD Biosciences), TNFα (200 ng/ml) (eBioscience) and LPS (1 µg/ml) (Sigma).

### Statistical Analyses

Data analyses were performed using GraphPad Prism Version 4.0 software. Data sets were tested for normal distribution using the Kolmogorov-Smirnov test. Parametric data was analyzed using T-tests or paired T-tests. Non-parametric data was compared using Mann-Whitney tests, Wilcoxon Matched Pairs tests, or Spearman correlations.

## Results

### Skewed ADCC-induced NK Cell Activation Profiles Mediated by Sera from HIV-infected Individuals

**Figure 1:**
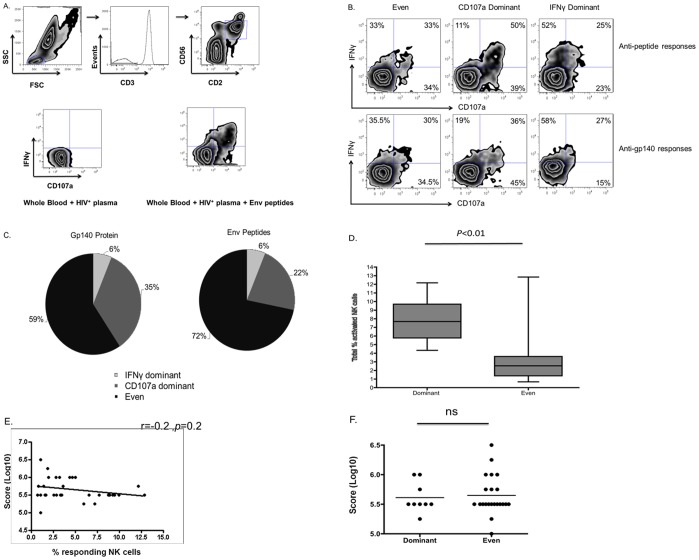
Differential NK cell activation patterns by HIV-specific ADCC. The ability of NK cells to respond to anti-HIV ADCC antibodies was assessed using a flow-based assay. (A) Stimulated cells were stained with fluorochrome conjugated antibodies against CD3, CD2, CD56, CD107a and IFNγ. After collection on a FACS II Canto, lymphocytes were gated upon and NK cells were identified as CD3^−^CD2^+^CD56^+^. Cells within the NK cell population were assessed for IFNγ production and CD107a expression prior to and following activation. (B) Zebra plots depict examples of the diverse anti-HIV ADCC responses obtained when NK cells from a common donor are stimulated with sera from different HIV-infected individuals in the presence of Env peptides (top) or gp140 protein (bottom). The numbers in the quadrants represent the percentages of responding NK cells that are mediating IFNγ^+^CD107a^−^, IFNγ^−^CD107a^+^ and IFNγ^+^CD107a^+^ responses. (C) The pie chart on the left illustrates the frequency with which IFNγ dominant, CD107a dominant and even response profiles were observed, when sera samples from 32 HIV-infected individuals were used to stimulate NK cells from a common donor in the presence of gp140 protein. The pie chart on the right depicts the same analysis for stimulation with Env peptides. (D) The box and whiskers plot depicts the assessment of the relationship between the ability of different sera to induce diverse anti-HIV ADCC functional profiles against HIV gp140 and the magnitude of the total ADCC response. The total percent of NK cell activation was compared between sera that induced “even” or “skewed” ADCC responses, using a T-test. (E) The scatter plot illustrates the relationship between the levels of sera anti-HIV gp140 IgG and the percent of total NK cells, from a common donor, activated by the sera in the whole blood ADCC assay in the presence of gp140. This correlation was assessed with the Spearman correlation. (F) The scatter plot depicts the assessment of the impact of the level of sera-associated anti-HIV gp140 IgG on the functional profile induced by different sera, evaluated by a T-test.

NK cells exhibit a number of functions when activated by the Fc portion of ADCC antibodies, including the expression of cytokines and the degranulation and lysis of target cells. We hypothesized that ADCC-induced NK cell effector functions may be differentially regulated depending on the cytokine milieu of the plasma. To evaluate this hypothesis we simultaneously evaluated sera samples obtained from a cohort of 32 antiretroviral therapy naive HIV-infected subjects for their ability to activate fresh NK cells in blood obtained from a single healthy donor in the presence of Env peptide or protein antigens. We utilized a previously described flow cytometric assay of antibody-mediated NK cell activation [12], [15], [29], [30], [32]–[34]. This simple assay studies the ability of donor NK cells within whole blood to be activated by antibodies within HIV^+^ sera samples recognizing HIV peptides or Env proteins. The NK cell activation in this assay occurs only when both peptides/proteins and antibodies are present (Fig. 1a). Furthermore, this assay is not dependent on NK cell recognition of immune complexes.

We observed marked differences in the ability of NK cells from the same donor to express IFNγ, CD107a or both effector molecules in response to the same HIV Env antigens using different HIV^+^ sera samples - examples are shown in Fig. 1b. We quantified this difference by assessing whether there was markedly greater IFNγ expression, greater CD107a expression or similar expression of both molecules from NK cells. Quantification was performed by using a 1.5% differential to determine if responses were skewed towards a particular response profile. As such, responses that exhibited equal to or higher than 1.5% NK cell expression of one effector molecule than the other were considered skewed for that response, while responses with less than 1.5% differences were considered equal. For stimulations involving gp140 protein CD107a was the predominant effector molecule expressed in 22% of samples, whereas in a minority (6% of samples) IFNγ expression predominated (Fig. 1c). Similarly, for stimulations involving Env peptide stimulation CD107a was the predominant effector molecule expressed for 35% of samples, whereas in a minority (6% of samples) IFNγ expression predominated (Fig. 1c). It should be noted that plasma samples that induce skewed responses appear to induce similarly skewed responses regardless of the source of donor NK cells. We assessed anti-HIV ADCC using a single plasma source on 20 HIV-uninfected controls. We found that the same source of plasma consistently induced a CD107a-skewed NK cell functional profile; the mean ratio between percent of NK cells expressing CD107a and percent of NK cells expressing IFNγ was 3.0 (range 1.2–16.9) across the 20 subjects.

Previous research has suggested that individual NK cells within an organism obtain diverse functional potentials through ontological processes, such as NK cell education. As such, we questioned if the differential responses induced by different serum samples were reflective of the activation of different arrays of functionally competent NK cells. To answer this question we compared the total percentage of activated NK cells present in individuals that mediated “skewed” and “even” ADCC responses against HIV gp140. This analysis demonstrated that skewed responses were associated with higher percentages of activated NK cells (7.9+/−0.8 vs. 3.4+/−0.6, *p*<0.01, Mann-Whitney test) (Fig. 1d). It should be noted that a similar observation was made for ADCC responses against HIV Env peptides (Data not shown).

Next, we evaluated if the differences in magnitude and functional profiles of NK cell-mediated ADCC responses to different plasmas could be explained by different levels of anti-HIV IgG present in the sera, or if it was related to other soluble sera factors. As such, an anti-Env IgG ELISA was utilized to assess the levels of anti-Env IgG in 31/32 sera samples (Due to limited sample availability). Figure 1E illustrates that no correlation was observed between the level of sera anti-Env IgG and the magnitude the anti-HIV ADCC response (r = 0.2, *p* = 0.2, Spearman correlation). Figure 1F illustrates that no difference in sera anti-Env IgG was observed between sera mediating “skewed” responses and those mediating “even” responses (*p*>0.05, Mann-Whitney test). These observations suggest that plasma factors in additional to Env-specific IgG levels are influencing the magnitude and functional profile of NK cell-mediated anti-HIV ADCC responses.

**Figure 2:**
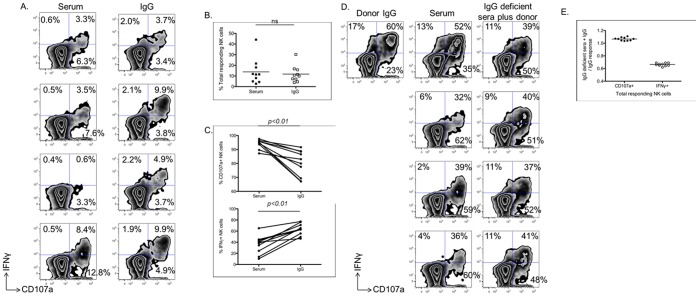
Effect of IgG purification on NK cell-mediated ADCC. The impact of non-IgG soluble sera factors was assessed by activating NK cells for ADCC functionality in the presence of whole sera, purified IgG, or common purified IgG in the presence of IgG-depleted sera from a series of donors. (A) Zebra plots depict the anti-HIV ADCC mediated by NK cells when incubated with whole sera or IgG purified from the same sera. The values depicted represent the percentages of total NK cells mediating CD107a^+^IFNγ^−^, CD107a^−^IFNγ^+^, or CD107a^+^IFNγ^+^ functional profiles. (B) The scatter plot depicts a comparison of the percentage of total NK cells mediating ADCC-induced effector functions after stimulation of NK cells from a common donor with sera or IgG purified from sera. This difference was assessed with a paired T-test. (C) The graph on the top illustrates the percent of responding NK cells expressing CD107a after stimulation with sera or IgG purified from the same sera. The graph on the bottom illustrates the percent of responding NK cells producing IFNγ after stimulation with sera or IgG purified from the same sera. Differences were assessed with paired T-tests. (D) Zebra plots depict the response of NK cells from a common donor to anti-HIV ADCC when incubated with purified IgG from a common donor, sera from a series of donors, or purified IgG from a common donor combined with IgG-depleted sera from the series of donors. The values depicted represent the percentages of responding NK cells mediating CD107a^+^IFNγ^−^, CD107a^−^IFNγ^+^, or CD107a^+^IFNγ^+^ functional profiles. (E) The graph illustrates the alteration of the NK cell-mediated ADCC functional profile induce by the common purified IgG after the addition of IgG-depleted sera from a series of donors.

Previous research has demonstrated that soluble plasma factors, such as cytokines, can alter the immune responsiveness of NK cells [16]. To assess if plasma-derived factors were responsible for these skewed ADCC responses, we next purified IgG from the sera of 10 HIV-infected subjects with predominantly CD107a expression profiles and assessed the Env-specific ADCC-induced activation profiles of NK cells activated with purified IgG in comparison to whole sera. Four representative samples from these experiments are shown in figure 2A. Despite having no effect on the total percentage of responding NK cells (*p*>0.05, Wilcoxon Matched Pairs Test) (Fig. 2B), IgG purification was consistently observed to alter the response towards a more IFNγ^+^ response profile (Fig. 2C). This is illustrated by the shift from plasma induced responses that consisted of 94% of activated NK cells expressing CD107a and 36% of activated NK cells producing IFNγ to pure IgG induced responses that consisted of 80% of activated NK cells expressing CD107a and 63% of activated NK cells producing IFNγ. There was significantly increased synthesis of IFNγ in ADCC mediated by purified IgG compared to that mediated by sera (63+/−3.3 vs. 36+/−5.1, *p* = 0.0001, paired T-test) and significantly decreased expression of CD107a in ADCC mediated by purified IgG compared to that mediated by sera (80+/−2.9 vs. 94+/−1.0, *p = *0.001, paired T-test). These results suggest that soluble factors, other than IgG, within sera, are affecting the cytokine expression/degranulation profile of NK cells following ADCC activation.

To confirm that the alteration of the NK cell responses towards higher IFNγ and lower CD107a expression following IgG purification was due to soluble factors present within the plasma, we next performed a set of experiments that involved separately adding IgG-depleted sera from 10 different HIV-infected donors that exhibited CD107a dominant responses to the purified IgG from a single donor. Four representative examples of these experiments are depicted in figure 2D. Similar to the results suggesting that ADCC responses induced by whole plasma are characterized by higher CD107a expression and lower IFNγ synthesis than ADCC responses mediated by purified IgG, the addition of IgG-depleted plasma from different donors to a common source of purified IgG increased the responding NK cells that expressed CD107a and decreased the responding NK cells that produced IFNγ (Fig. 2E). Cumulatively, these results demonstrate that soluble factors other than IgG, which are present within the plasma/sera of HIV-infected individuals, influence the functional profile of NK cells mediating anti-HIV ADCC.

### Influence of Cytokines on HIV-specific ADCC Activation of NK Cells

**Figure 3:**
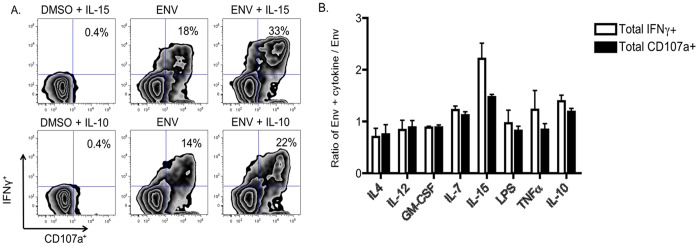
Effect of exogenous cytokines on ADCC-induced NK cell activation. A suite of 7 cytokines and LPS was added separately to a mix of HIV^+^ plasma co-incubated with healthy donor blood and HIV-1 Env antigens. Gated CD3^−^CD2^+^CD56^+^ NK cells were studied for CD107a and IFNγ expression. These experiments were repeated using whole blood from four separate donors (A) Zebra plots depict the anti-HIV ADCC mediated by NK cells when incubated with cytokines in the absence of anti-HIV antibodies and Env antigens, incubated with anti-HIV antibodies and Env antigens with no cytokines added, or with the addition of cytokines. The values shown represent the percentages of total NK cells expressing both IFNγ and CD107a. (B) The effect of all cytokines and LPS on ADCC-driven NK cell activation is shown, expressed as a ratio of the response with cytokine added to the response without cytokine. The effect of cytokine addition on both total CD107a^+^ and total IFNγ^+^ responses is shown. Error bars represent variation between different whole blood donors.

As our initial results suggested that soluble plasma factors have an impact on NK cell-mediated effector functions, we selected a series of soluble factors to assess their influence on the anti-HIV ADCC induced activation profiles of NK cells. As discussed in the introduction, these factors were chosen due to their previously observed relevance to HIV infection [18]–[27]. These factors were added into a standard culture of healthy donor whole blood, Env antigens and plasma from an HIV-infected donor with known anti-Env ADCC antibodies. This experiment was repeated with whole blood from four HIV-uninfected donors. We observed consistent alterations in the profile of NK cell activation in the presence of several cytokines (Figure 3). GM-CSF and IL-4 both exhibited an inhibitory effect on overall Env-specific ADCC-induced NK cell activation, decreasing responses in all donors studied. IL-12, TNF-α and IL-7 had little overall effect on ADCC-mediated NK cell activation in the donors. However, both IL-10 and IL-15 had a markedly positive effect on ADCC-induced NK cell activation, increasing ADCC responses in all 4 donors. These results suggest that cytokines can alter the immunological microenvironment within which anti-HIV ADCC responses occur, increasing or decreasing the potency of these anti-viral responses.

### Effects of IL-10 and IL-15 on Anti-HIV ADCC Induced NK Cell Activation

**Figure 4:**
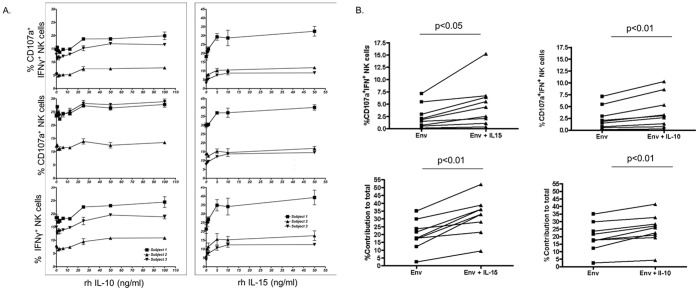
Consistent effects of IL-10 and IL-15 across numerous donors. (A) Titration curves depict the effects of adding IL-10 or IL-15 to donor NK cells from three donors, which were all stimulated with the same HIV^+^ plasma in the presence of HIV-1 Env antigen. (B) NK cells from a common donor were stimulated with plasma from 9 HIV-infected individuals in the presence of HIV-1 Env antigen, with or without exogenous cytokines. Graphs on the top demonstrate the consistency of the effect of exogenous IL-10 and IL-15 on bifunctional (IFNγ^+^CD107a^+^) response profiles, regardless of the source of the plasma. Graphs on the bottom depict the consistency of the effect of exogenous IL-10 (50 ng/ml) and IL-15 (5 ng/ml) on the percentage contribution bifunctional responses make to the total NK cell response.

As IL-10 and IL-15 both enhanced NK cell-mediated anti-HIV ADCC effector functions, we performed a detailed assessment of the influences of these cytokines on NK cell-mediated anti-HIV ADCC. Since we observed an impact of these cytokines on the ability of NK cells to both produce IFNγ and express CD107a, we first performed titrations to elucidate the concentrations at which these cytokines are active (Figure 4A). These titrations also served to confirm the consistency of the effect of IL-10 and IL-15 across NK cells from several donors. While IL-10 required relatively high levels (i.e., 25–50 ng/ml) to enhance anti-HIV ADCC induced NK cell IFNγ production and degranulation, IL-15 maximally increased both NK cell effector functions at low concentrations (i.e., 5 ng/ml). These results demonstrate that cytokines can influence the effector functions of NK cells at concentrations that could be achievable through therapeutic mechanisms.

Control of viral infections has been associated with simultaneous mediation of several cell-based effector functions [35]–[37]. Indeed, natural control of HIV-infection has been associated with polyfunctional CTL and NK cell responses. As polyfunctional, rather than monofunctional, NK cell-mediated responses would be preferable to obtain by prophylactic and/or therapeutic interventions, we next assessed the impact of exogenous cytokines on the ability of NK cells to mediate more than one effector function simultaneously. Since different plasmas contain different arrays of biologically active cytokines that could influence the effects of exogenous cytokines, we assessed if the effects of adding IL-10 and IL-15 were consistent across a series of HIV-infected plasmas. Regardless of the plasma used in the whole blood ICS assay, we observed a consistent increase in the frequency of cells capable of mediating bifunctional (IFNγ^+^CD107a^+^) responses when IL-10 (2.5+/−0.8 vs. 3.9+/−1.2, *p*<0.01, paired T-test) and IL-15 (2.5+/−0.8 vs. 4.9+/−1.5, *p*<0.05, paired T-test) were added (Figure 4B). Indeed, exogenous IL-10 (19.7+/−3.2 vs. 24.8+/−3.4, *p*<0.01, paired T-test) and IL-15 (19.7+/−3.2 vs. 32.0+/−3.9, *p*<0.01, paired T-test) increased the contribution that bifunctional responses made to the total NK cell response (Figure 4B). The observation that exogenous cytokines have consistent effects across a series of HIV-infected plasmas suggests that therapeutic use of exogenous cytokines would have a similar effect across many HIV-infected individuals. Overall, these results suggest that polyfunctional cellular responses, which have been associated with natural protection from HIV disease progression [1], [35]–[37], are achievable through anti-HIV ADCC antibodies, especially in the presence of exogenous cytokines.

### Importance of NK Cell Education for Effects of Exogenous Cytokines on NK Cell-mediated Anti-HIV ADCC

**Figure 5:**
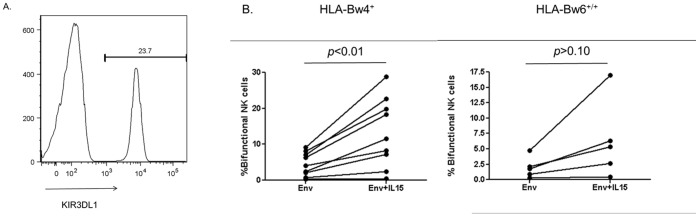
Role of NK cell education in exogenous cytokine stimulation. Whole blood from nine HLA-Bw4 carriers and five HLA-Bw6 homozygous healthy controls was incubated with ADCC-competent plasma in the presence of HIV-1 Env antigen, with or without exogenous IL-15. (A) CD3^−^ CD56^+^ NK cells were gated upon and assessed for KIR3DL1 expression. (B) KIR3DL1^+^ NK cells from HLA-Bw4 carriers and HLA-Bw6 homozygotes were assessed for their ability to mediate bifunctional (i.e., CD107a^+^IFNγ^+^) anti-HIV ADCC responses in the absence and presence of IL-15. The graph represents the influence of IL-15 on the bifunctionality of NK cells from the KIR3DL1^+^ subset in both groups. The impact of IL-15 on the bifunctionality of these NK cells was assessed using paired T-tests.

The ability of NK cells to mediate effector functions is controlled by a developmental process known as education [28]. In short, this process ensures self-tolerance of NK cells by only conferring functional potential upon NK cells that express inhibitory receptors for self-ligands. Recent research suggests that the stronger the inhibitory signal delivered to NK cells during this process, the stronger and wider array of effector functions mediated by the NK cell [38]. Indeed, polyfunctional NK cell responses occur more frequently in NK cells that are educated by KIR3DL1/HLA-Bw4 combinations that are protective against HIV infection and/or disease progression [36], [37]. Interestingly, KIR3DL1^+^ NK cells from HLA-Bw4 carriers have also been demonstrated to mediate higher bifunctional anti-HIV ADCC responses than the same NK cell subset from HLA-Bw6 homozygotes [15]. As we observed a greater effect of exogenous cytokines on bifunctional NK cells, we next assessed if NK cells educated through KIR3DL1/HLA-Bw4 interactions are more sensitive than KIR3DL1^+^ NK cells from HLA-Bw6 homozygotes to exogenous cytokine treatment. Figure 5a illustrates the gating strategy we employed to distinguish KIR3DL1^+/−^ NK cells. Since exogenous IL-15 induced the largest increase in bifunctional ADCC responses, we assessed the role of educated NK cells in exogenous cytokine enhanced functionality using IL-15. We assessed groups of 9 HLA-Bw4^+^ individuals and 5 HLA-Bw6 homozygous donors and found that exogenous IL-15 treatment significantly increased the bifunctional activity of KIR3DL1^+^ NK cells from HLA-Bw4 carriers (4.5+/−1.1 vs. 13.3+/−3.2, *p*<0.01, paired T-test), but not from HLA-Bw6 homozygotes (1.98+/−0.8 vs. 6.4+/−2.8, *p*>0.05, paired T-test) (Figure 5B). Although increases in bifunctional activity were consistently observed HLA-Bw6 homozygotes, the reason these changes did not reach significance could reflect the lower responsiveness of non-educated NK cells to cytokine stimulation [14], [39].

## Discussion

Several studies have linked NK cell-mediated anti-HIV ADCC to protection against HIV infection and/or disease progression [6]–[10]. Although several assays exist to evaluate anti-HIV ADCC, the readouts of these experimental systems are influenced by several factors that could confound associations of ADCC with HIV disease progression. Most assays measuring anti-HIV ADCC analyze plasma or sera from HIV-infected individuals. As HIV infection is associated with immune activation and perturbations of plasma cytokine levels [16], these samples often contain a suite of biologically active factors that can skew the ADCC measurement. Indeed, using a NK cell activation anti-HIV ADCC assay we observed that, depending on the source of serum used, NK cells from a common donor mediated a series of responses either skewed towards cytokine production, degranulation or equally spread across both effector functions. These skewed responses were dependent upon soluble plasma/sera factors and not solely due to intrinsic qualities of anti-HIV antibodies, as purification of IgG was associated with alteration of NK cell effector functions. Exogenous addition of IL-10 or IL-15 markedly increased NK cell-mediated anti-HIV ADCC effector functions. The effects of IL-15 were observed most potently on NK cells educated through co-expression of KIR3DL1 and HLA-Bw4, which have previously been associated with protection against HIV infection and/or disease progression [40], [41]. Of the molecules tested, IL-4 and GM-CSF had a negative influence on NK cell activation, and IL-4 has been implicated in HIV pathogenesis [18]. Surprisingly, no effect was observed for IL-12 on anti-HIV ADCC. This could reflect differences in the methodology utilized in the current manuscript compared to other investigations. For example, the current investigation added IL-12 to the NK cells for a brief five hour period. Other investigators generally add IL-12 to the NK cells for an overnight period prior to NK cell functional assays [42].

The effects of cytokines on NK cell-mediated anti-HIV ADCC effector functions are potently observed within the KIR3DL1 educated NK cell subset mediating both IFNγ production and degranulation as assessed by CD107a expression. As educated NK cells mediating polyfunctionality have been associated with protection from HIV infection and/or disease progression [36], [37], the ability of cytokines to alter the functionality of these NK cells may partially explain the association of certain cytokine perturbations with protection from HIV infection or advancement of HIV disease. Indeed, the functional observations of this study suggest this possibility. We observed that IL-10 and IL-15 enhanced NK cell functionality. Previous research has demonstrated that both IL-10 and IL-15 can enhance NK cell functions, including ADCC [14], [43]–[48]. The current investigation, however, has built upon these previous studies by investigating the role for IL-15 in a novel assay, as well as the ability of this cytokine to improve upon the functions of “educated” and “uneducated” NK cells. Interestingly, IL-15 has been demonstrated to be increased in the breast milk that is fed to exposed uninfected infants [49]. Meanwhile, exposed-uninfected individuals and slow progressing individuals have been demonstrated to carry IL-10 promoter polymorphisms that result in high levels of plasma IL-10 [50], [51]. Although IL-10 and IL-15 could act through multiple mechanisms to assist in control of HIV, we speculate that an important mechanism could be their ability to enhance NK cell responsiveness to ADCC antibodies. Future studies should evaluate the association of overlap between NK cell education and plasma cytokine levels with protection from HIV infection.

The observation that IL-10 and IL-15 can enhance anti-HIV NK cell activity suggests that these cytokines may be potential resources for prophylactic and/or therapeutic interventions. Although the concentrations of exogenous cytokines that are required to observe increases in NK cell functionality are above what is observed *in vivo* [52], it is worth noting that long-term changes in *in vivo* concentrations of cytokines could still be mediating the skewed responses mediated by several plasma samples. Previous research has demonstrated that the effects of cytokines are synergistic [53]. Furthermore, neutralization of plasma cytokines has been demonstrated to rescue NK cells from the effects of inhibitory endogenous cytokines, such as TGF-β [54]. Lastly, although the high concentrations of exogenous cytokines required to observe NK cell activation may not reflect *in vivo* concentrations, the concentrations may still be safely utilized for preventative or therapeutic interventions. The levels of IL-15 required to activate NK cells to mediate anti-HIV ADCC are particularly intriguing, as it may be feasible to therapeutically administer similar levels of IL-15 to HIV-infected individuals [55]. Although, the levels of exogenous IL-10 required to stimulate increased anti-HIV ADCC are much higher, they too could likely be supplied safely from an exogenous source [56]. The fact that IL-10 enhances NK cell-mediated anti-HIV ADCC, however, makes it a particularly attractive target. Although some previous research has associated IL-10 with poor viral control, this is mostly due the detrimental effects of IL-10 on CTL [19]. Future research should investigate IL-10 as a component of an antibody containing anti-HIV microbicide that utilizes the ADCC effector function of NK cells. Our results, in combination with previous demonstrations of high-producing IL-10 promoter polymorphisms in exposed uninfected individuals [50], suggest that IL-10 may be particularly well-suited to protect against HIV infection.

Demonstrating that exogenous cytokines can enhance NK cell-mediated anti-HIV ADCC responses that have been associated with protection from HIV infection and/or disease progression is an important step towards understanding how to design better ADCC-based therapeutics or vaccines. Our data suggests vaccines co-expressing IL-15 [57] could result in strongly enhanced ADCC potency. While this study investigated the effect of exogenous cytokines on a small number of NK cell effector functions, future research should elucidate the effector functions involved in viral suppression/control and evaluate the effects of exogenous cytokine stimulation on these functions. Additionally, analysis of monoclonal ADCC antibodies could also evaluate the influence of Fc region glycosylation patterns on NK cell activation. With a broader understanding of the factors determining the potency of anti-HIV ADCC responses, such as NK cell education, antibody specificity and soluble plasma factors, enhanced ADCC-based therapies and vaccines can be more rationally designed.
